# Elimination of negative feedback in TLR signalling allows rapid and hypersensitive detection of microbial contaminants

**DOI:** 10.1038/s41598-021-03618-9

**Published:** 2021-12-24

**Authors:** Clovis H. T. Seumen, Urte Tomasiunaite, Daniel F. Legler, Christof R. Hauck

**Affiliations:** 1 Lehrstuhl für Zellbiologie, Fachbereich Biologie, Maildrop 621, Universität Konstanz, Universitätsstrasse 10, 78457 Konstanz, Germany; 2 Kooperatives Promotionskolleg “InViTe”, Universität Konstanz, 78457 Konstanz, Germany; 3 Biotechnology Institute Thurgau (BITg), University of Konstanz, Kreuzlingen, Switzerland; 4 Konstanz Research School Chemical Biology, Universität Konstanz, 78457 Konstanz, Germany

**Keywords:** Cell biology, Immunology

## Abstract

The exquisite specificity of Toll-like receptors (TLRs) to sense microbial molecular signatures is used as a powerful tool to pinpoint microbial contaminants. Various cellular systems, from native human blood cells to transfected cell lines exploit TLRs as pyrogen detectors in biological preparations. However, slow cellular responses and limited sensitivity have hampered the replacement of animal-based tests such as the rabbit pyrogen test or lipopolysaccharide detection by Limulus amoebocyte lysate. Here, we report a novel human cell-based approach to boost detection of microbial contaminants by TLR-expressing cells. By genetic and pharmacologic elimination of negative control circuits, TLR-initiated cellular responses to bacterial molecular patterns were accelerated and significantly elevated. Combining depletion of protein phosphatase PP2ACA and pharmacological inhibition of PP1 in the optimized reporter cells further enhanced the sensitivity to allow detection of bacterial lipoprotein at 30 picogram/ml. Such next-generation cellular monitoring is poised to replace animal-based testing for microbial contaminants.

## Introduction

Innate immune responses are tightly regulated to effectively defend against infectious organisms, while minimizing damage to host cells and tissues. Innate immune responses are induced upon engagement of cellular sensors collectively termed pattern recognition receptors (PRRs)^1^. PRRs encompass NOD-like receptors, RIG-like receptors, cGAS/STING, and Toll-like receptors (TLRs) and are mainly expressed by immune cells, where they mediate detection of diverse microbial molecular signatures, also known as pathogen-associated molecular patterns (PAMPs)^2–5^. Though individual PRRs have evolved to respond to specific PAMPs, their stimulation triggers common downstream signaling pathways that impinge on the activation of characteristic transcription factors^6^. Nuclear factor kappa-light chain enhancer of activated B-cells (NF-κB) is the paradigm transcription factor activated upon PAMP detection and the main driver behind the TLR-initiated de novo expression of pro-inflammatory cytokines^7^. As a consequence, NF-κB transcriptional activity sparks inflammation accompanied by elevated body temperature or fever^8^. Accordingly, microbial components sensed by TLRs are potent pyrogens and PAMP contamination is a major safety concern for parenteral solutions and medical devices. To assure the absence of pyrogens, different testing regimes have been implemented for surveillance of microbial contamination in medical manufacturing. While the use of the rabbit pyrogen test (RPT) and the Limulus amoebocyte lysate (LAL) are controversial because of the sacrifice of a large number of animals and due to inherent differences between distantly related species^9^, human cell-based assays also have limitations. The most widely employed animal-free alternative test is the monocyte activation test (MAT). The MAT exploits the intrinsic capability of primary human monocytes or monocytoid cell lines to respond to various PAMPS with the expression of immune mediators, such as TNFα or IL-6, which serve as a readout for TLR stimulation^10–14^. Though several approaches, such as overexpression of TLRs or their co-receptors in human cell lines, have been put forward to reduce the dependence on fresh human blood samples and to increase the sensitivity of the MAT, all these cell-based approaches require prolonged incubation times of > 20 h to reach their endpoint^12,15^.

Given the fact that TLR signaling heavily depends on ubiquitination, selective protein–protein interaction, and protein phosphorylation^16–19^, we wondered if the signaling output of this pathway could be accelerated and elevated by diminishing counter-regulatory processes. For example, the suppressor of cytokine signaling 1 (SOCS1) is an integral component of a negative feedback loop to dampen TLR responses^20^. In this regard, the SH2-domain of SOCS1 binds to the TLR receptor-associated adaptor molecule Mal/TIRAP, which is tyrosine phosphorylated by BTK upon TLR stimulation^21–23^. SOCS1 binding drives the polyubiquitination of Mal, thereby promoting its proteasomal degradation and limiting TLR signaling^24^.

To put our idea to the test, we performed genetic and pharmacologic manipulation of negative regulators of the TLR pathway in a monocytic cell line. Here, we report that deletion of SOCS1 sensitizes cells to microbial PAMPs and thereby strongly elevates and accelerates signaling output of an established NF-κB reporter system. Based on these results, we devise an optimized monocytic cell line, called H-17, where depletion and inhibition of specific protein phosphatases results in faster and more sensitive responses towards agonists of TLR1/TLR2, TLR2/TLR6, TLR5, and TLR9. In the final configuration, the optimized reporter cells were able to flag the presence of picomolar amounts of bacterial lipoproteins within 3 h of exposure surpassing available systems in speed and sensitivity. Genetically optimized reporter cells could function as next generation cellular systems to monitor microbial contaminants.

## Results

### A blue fluorescent protein reporter system for NF-κB activity

**Figure 1:**
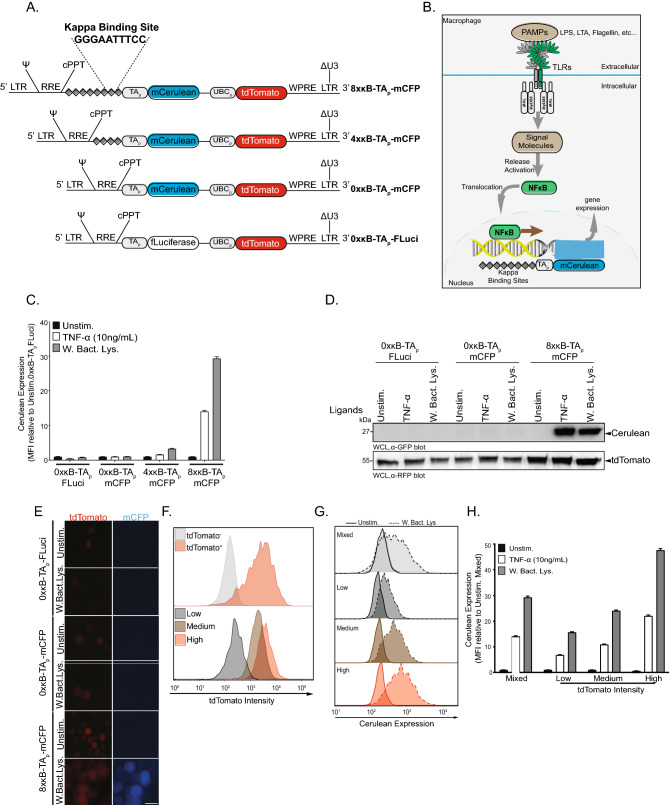
A blue fluorescent protein reporter system for NF-κB activity. (**A**) Chart of the core features of the lentiviral constructs used to generate the transcriptional reporter system and the kappa consensus binding site. (**B**) Schematic description of TLR-mediated NF-κB activation in macrophages and the central principle of a blue fluorescent protein reporter system. (**C**) Quantification of mCFP in THP-1 cells expressing the four constructs illustrated above and stimulated with TNF-⍺ (10 ng/ml) and *E. coli* whole lysate (W. Bact. Lys.) for 24 h respectively. Bar graphs show median fluorescent intensity (MFI) ± SEM (n = 4). (**D**) Western blots showing relative levels of mCFP and tdTomato in THP-1 expressing three constructs after stimulation as above. See also Supplementary Fig. S1A for uncropped, full-size original Western Blot. (**E**) Representative fluorescent microscopy images of designer cells expressing tdTomato (Red) and mCFP (Blue) stimulated as above. Scale bar = 10 µm. (**F**) Representative flow cytometry histograms for tdTomato level in transduced THP-1 cells (Red) versus wildtype (grey; upper panel) and the sorting according to low, medium, and high tdTomato intensity (lower panel). Histograms are normalised to the mode. (**G**) Representative flow-cytometry histograms of mCFP in mixed THP-1 cells (light grey), low (dark grey), medium (brown) and high (red) tdTomato-expressing THP-1 cells, respectively. Histograms are normalised to the mode. (**H**) Quantification of mCFP in cells from (**G**). Bar graphs show MFI ± SEM (n = 4). See also Fig. S1.

The transcription factor NF-κB is a central component in regulating the inflammatory response by inducing genes encoding type I interferon and pro-inflammatory cytokines^25^. NF-κB contains a well-preserved DNA binding domain that recognizes a consensus DNA sequence (called a Kappa binding site) in the promoter region of NF-κB–regulated genes^26^. In order to generate a cellular reporter system, three constructs containing 0, 4, or 8 disjunct kappa binding sites upstream of the coding sequence for the monomeric blue-fluorescent protein cerulean (mCFP) were stably introduced into monocytic THP-1 cells to allow the monitoring of TLR-induced mCFP expression (Fig. 1A,B). Furthermore, a control construct encoding the promoterless firefly luciferase (FLuciferase) was also prepared. All constructs encompassed a second independent expression cassette driving constitutive expression of the red fluorescent protein tdTomato (Fig. 1A). When the cells were challenged with *E. coli* lysate, a significantly higher level of mCFP was observed in 8xκB-TAp-mCFP cells compared to the 4xκB-TAp-mCFP cells (30- versus fourfold increase, respectively) upon stimulation for 24 h (Fig. 1C,D; Supplementary Fig. S1A). Similarly, TNF-α (10 ng/mL) induced the highest level of mCFP expression in 8xκB-TAp-mCFP, with ~ 16-fold increase after 24 h (Fig. 1C,D; Supplementary Fig. S1A). These results demonstrate that the magnitude of the reporter gene expression and subsequently the activation of NF-κB in the reporter cells correlates with the number of the kappa binding sites upstream of the reporter gene. Furthermore, no background expression of mCFP was detected in THP-1 cells, where the control constructs 0xκB-TAp-mCFP and 0xκB-TAp-FLuci were introduced, verifying that mCFP expression and enhanced fluorescence was strictly dependent on stimulus-induced NF-κB activation (Fig. 1C,D; Supplementary Fig. S1A). The increased expression of mCFP as detected by Flow cytometry and Western Blotting (Fig. 1C,D; Supplementary Fig. S1A), was also verified by fluorescence microscopy, which demonstrated that 8xκB-TAp-mCFP cells displayed the strongest response to the stimulus (Fig. 1E). Interestingly, expression of the tdTomato protein was heterogenous in the cells indicating that differences in the number and chromosomal location of retroviral integration sites might influence the signaling output of individual cells (Fig. 1E). Therefore, we exploited tdTomato expression to sort the stably transduced 8xκB-TAp-mCFP THP-1 cell population into low, medium, and high tdTomato-expressing subpopulations (Fig. 1F). Stimulation of these distinct cell populations revealed that high tdTomato-expressing cells did not show increased background expression of the mCFP reporter, but rather responded with a strongly elevated magnitude (~ 46-fold induction of mCerulean expression in high tdTomato expressing cells vs. ~ 25-fold or ~ 17-fold in medium or low expressing 8xκB-TAp-mCFP THP-1 cells, respectively) upon stimulation with *E. coli* whole bacterial lysate or with TNF-α (Fig. 1G,H). In contrast to whole bacterial lysates or isolated bacterial lipoprotein (Pam3CSK4), fungal β-glucan or mycoplasma did not elicit strong reporter activity, pointing to a selective recognition of PAMPs by THP-1 cells (Supplementary Fig. S1B-D). Nevertheless, these results indicated that the generated 8xκB-TAp-mCFP THP-1 cells can serve as a robust NF-κB reporter system, but also suggested that the cellular response might be even stronger in individual clones amongst the high tdTomato expressing cells.

### Individual reporter cell clones show elevated responses to TLR agonists

**Figure 2:**
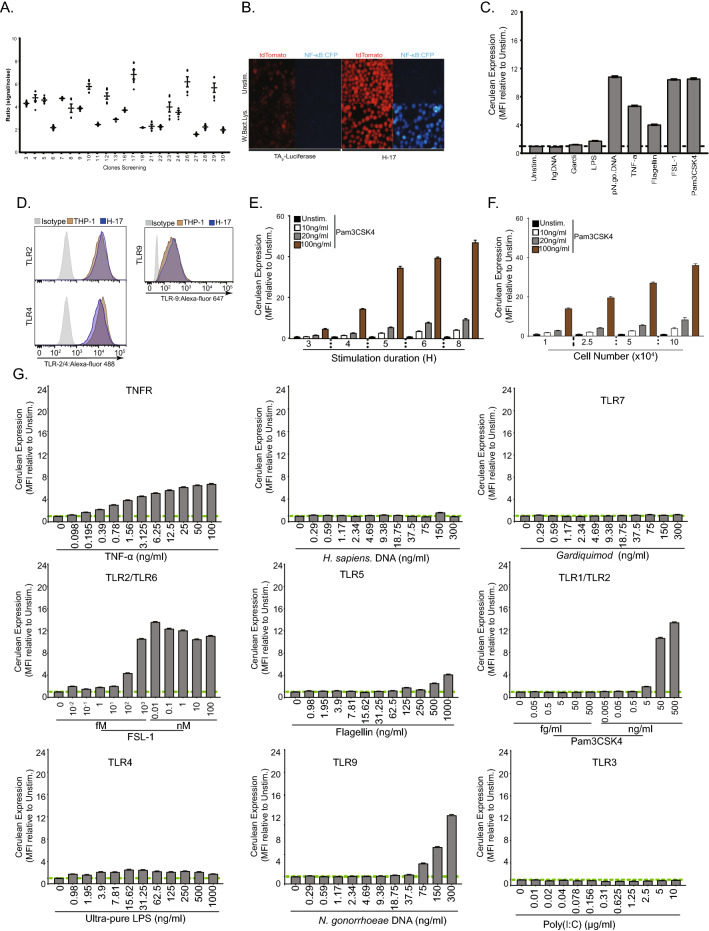
Individual reporter cell clones show elevated responses to TLR agonists. (**A**) Signal to noise ratio response of individual reporter cell clones after stimulation with Pam3CSK4 (100 ng/ml) for 6 h. (**B**) Representative fluorescent microscopy images of H-17 expressing tdTomato (Red) and mCFP (Blue) stimulated as above. Scale bar = 10 µm. (**C**) Quantification of mCFP in H-17 cells in response to human genomic DNA, gardiquimod, LPS, piliated *N. gonorrhoeae* genomic DNA, TNF-⍺, *B.subtilis* Flagellin, FSL-1 and Pam3CSK4 compare to unstimulated cells. Bar graphs represent MFI ± SEM (n = 4). (**D**) Representative flow cytometry Dot plots for Toll like receptors (2, 4 and 9) from WT and H-17 and percentage of positive cells are indicated on each individual plots. Histograms are normalised to the mode. (**E**) Quantification of mCFP expression kinetic in H-17 upon stimulation of Pam3CSK4 after 3, 4, 5, 6 and 8 h. Bar graphs represent MFI ± SEM (n = 4). (**F**) Quantification of mCFP expression with different cell number 1, 2.5, 5 and 10 * 10^4^ cells per well respectively. Bar graphs represent MFI ± SEM (n = 4). (**G**) Quantification of the H-17 limit of detection of specific TLR agonist TNF-α, H. sapiens genomic DNA, Gardiquimod, Poly(I:C), CpG ODN, FSL-1, Flagellin, Pam3CSK4 and *N. gonorrhoeae* genomic DNA respectively. Bar graphs represent MFI ± SEM (n = 3). See also Fig. S2.

To determine, if high dTomato expressing 8xκB-TAp-mCFP THP-1 cells show improved responses to TLR stimulation, we isolated single high dTomato-expressing cells by fluorescence-activated cell sorting and developed clonal cell lines. Upon stimulation with *E. coli* whole bacterial lysate, several clones showed elevated mCFP expression (Fig. 2A). In particular, clone H-17 exhibited the strongest increase of mCFP fluorescence, which was also readily apparent in microscopic images (Fig. 2A,B). Clone H-17 cells showed a strong response towards various known microbial molecular signatures. For example, FSL-1 (100 nM), a TLR2/6 ligand, and Pam3CSK4 (100 ng/mL), a TLR1/2 ligand, both caused a ninefold increase in mCFP expression, flagellin (0.5 µg/mL), a TLR5 ligand, led to a ~ 4 increase, and gonococcal genomic DNA as a TLR9 ligand resulted in ~ tenfold increased expression of mCFP (Fig. 2C). In contrast, ultra-pure lipopolysaccharide (LPS), and Gardiquimod, which should stimulate TLR4 and TLR7, respectively, did not increase NF-κB reporter activity in clone H-17 (Fig. 2C). Besides TLR2 and TLR9, TLR4 was clearly expressed by the 8xκB-TAp-mCFP THP-1 parent cell line as well as by clone H-17 (Fig. 2D), suggesting that these cells apparently lack other components needed for TLR4-mediated LPS detection. Using the TLR1/2 ligand Pam3CSK4 as a potent stimulus, H-17 cells showed a dose-dependent increase in mCFP fluorescence, which was readily detectable as early as 3 h after stimulation (Fig. 2E). Moreover, the magnitude of the signal output by clone H-17 also depended on the cell density seeded in the 96-well plate, with 5 × 10^4^–1 × 10^5^ cells/well giving the highest response (Fig. 2F). To identify the limit of detection (LOD), which corresponds to the lowest concentration of agonist giving a significant signal above the background observed for unstimulated cells, clone H-17 cells were stimulated for 6 h with increasing concentrations of different ligands (Fig. 2G). While H-17 cells again failed to respond to TLR3, TLR4, and TLR7 stimulation, enhanced expression of mCFP was already detectable at 0.1 fM FSL-1 (TLR2/TLR6), 5 ng/ml Pam3CSK4 (TLR1/TLR2), 125 ng/ml flagellin (TLR5), and 75 ng/ml of bacterial genomic DNA (TLR9) (Fig. 2G). Human genomic DNA or DNAse-treated bacterial genomic DNA did not elicit a response confirming that these DNA preparations did not contain other stimulating bacterial products (Fig. 2G and Supplementary Figure S2). Collectively, these findings demonstrated that clone H-17 cells can be used as a reporter for several microbial contaminants with extremely high sensitivity towards TLR2 agonists.

### SOCS1 deletion lowers the detection limit of clone H-17 cells

**Figure 3:**
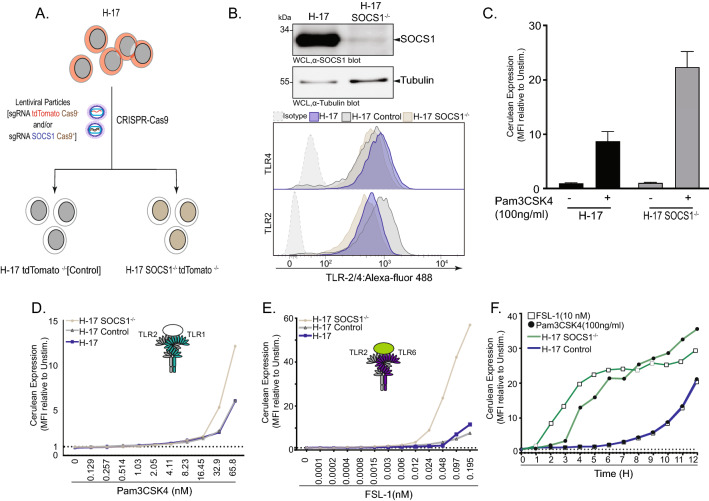
SOCS1 deletion lowers the detection limit of clone H-17 cells. (**A**) Graphs depicting CRISPR/Cas-9-mediated SOCS1^−/−^ and targeting strategy for SOCS1 exon 1. (**B**) Western blots showing that SOCS1 was absent in H-17 SOCS1^−/−^ cells compare to H-17 cells [upper panel]. See also Supplementary Fig. S3B for uncropped, full-size original Western Blot. Representative flow cytometry histograms for TLR2 and TLR4 on H-17, H-17 Mock and H-17 SOCS1^−/−^ cells. Percentage of positive cells is indicated on corresponding panels. Histograms are normalised to the mode [lower panel]. (**C**)**.** Quantification of mCFP expression in H-17 SOCS1^−/−^ clone 3. Bar graphs represent MFI ± SEM. (**D-E**) Quantification of H-17 SOCS1^−/−^ [brown] response to different concentrations of Pam3CSK4 (**D**) or FSL-1 (**E**) compared to H-17 [blue] and H-17 Control [gray] respectively. Bar graphs represent MFI ± SEM. (**F**)**.** Quantification of mCFP expression kinetic in H-17 SOCS1^−/−^ [green line] and H-17 cells [blue line] upon stimulation with Pam3CSK4 (100 ng/ml) [filled circles] and FSL-1 (10 nM) [open squares] for 0–12 h. Line curves represent MFI.

We speculated that the magnitude of reporter gene activity in clone H-17 cells might be further augmented by depriving these cells of negative regulators of TLR signaling. In this context, the suppressor of cytokine signaling 1 (SOCS1) is involved in a negative feedback loop to curb TLR signaling by inducing the ubiquitination and proteasomal degradation of key TLR associated proteins such as Myd88-Adaptor-Like (Mal, also called TIRAP). To this end, we disrupted the *SOCS1* gene via CRISPR-Cas9 (Fig. 3A). To facilitate identification of CRISPR-modified cells, we targeted not only SOCS1 with a specific sgRNA, but also applied simultaneously a second sgRNA targeting the tdTomato expression cassette present in H-17 cells. Accordingly, the disappearance of tdTomato expression indicates transduced cells with productive expression of Cas9 and was used as a marker to enrich for tdTomato/SOCS1-double-deficient clones (H-17 SOCS1^−/−^; Fig. 3A and Supplementary Figure S3A). In addition, we generated a control cell line, where only the tdTomato-specific sgRNA was applied, so that only dTomato expression was disrupted (H-17 Control; Fig. 3A and Supplementary Figure S3A). As verified by Western blotting, SOCS1 expression was completely absent H-17 SOCS1^−/−^ before and after stimulation with Pam3CSK4 (Fig. 3B and Supplementary Figure S3B). In H-17 SOCS1^−/−^ cells the expression of TLR2 and TLR4 was slightly reduced compared to the parental H-17 cells (Fig. 3B). However, the H-17 SOCS1^−/−^ cells showed more than a doubling of mCFP expression in response to the synthetic lipoprotein Pam3CSK4 (Fig. 3C,D). This increased responsiveness of the H-17 SOCS1^−/−^ cells translated into a significantly lower detection limit for Pam3CSK4 (2 ng/ml) and FSL-1 (12 pM) compared to the H-17 Control cells and H-17 parent cell line (Fig. 3D,E). Furthermore, the response of H-17 SOCS1^−/−^ cells towards Pam3CSK4 (100 ng/mL) or FSL-1 (10 nM) could be observed already 3 h after stimulation, while reporter gene accummulation in the H-17 control cells was only evident after 9–10 h (Fig. 3F). These results provided proof-of-concept that genetic manipulation of negative regulators of TLR signaling can lead to a gain of function phenotype with regard to stimulus-dependent NFκB activation without increasing background signaling activity.

### PP2ACA depletion sensitizes H-17 cells and reduces their response time towards lipoproteins

**Figure 4:**
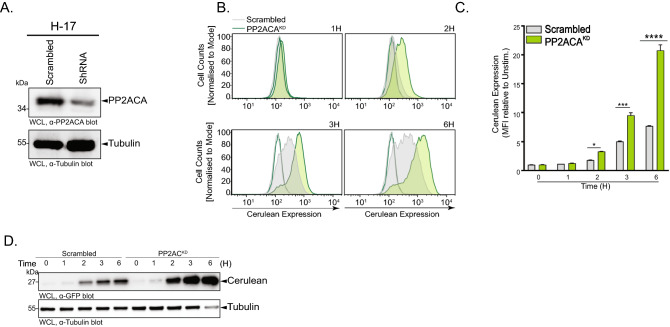
PP2ACA depletion sensitizes H-17 cells and reduces their response time towards lipoproteins. (**A**) Western blots showing PP2ACA depletion in H-17 PP2ACA^KD^ compare to scrambled H-17 cells. See also Supplementary Fig. S4A for uncropped, full-size original Western Blot. (**B**) Representative flow cytometry histograms of mCFP expression in H-17 PP2ACA^KD^ [green] compared to scrambled H-17 [gray] after treatment for 1, 2, 3, or 6 h with Pam3CSK4 (100 ng/ml). Histograms are normalised to the mode. Unfilled histograms represent unstimulated cells. (**C**) Quantification of mCFP expression in H-17 PP2ACA^KD^ compared to scrambled H-17 after treatment for 1, 2, 3, or 6 h with Pam3CSK4 (100 ng/mL). Bar graphs represent MFI ± SEM (n = 4). (**D**) Western blots showing increasing expression of mCFP in H-17 PP2ACA^KD^ compared to Scrambled H-17 after treatment with Pam3CSK4 (100 ng/ml) for 1, 2, 3 or 6 h. See also Supplementary Fig. S4B for uncropped, full-size original Western Blot.

Though SOCS-1 is a negative regulator of TLR signaling, it is a transcriptional target of NF-κB and only comes into play upon prolonged stimulation of the pathway^27^. We wondered, if it is possible to enhance the sensitivity and further accelerate the response of H-17 cells to TLR agonists by modifying an integral component of the TLR-induced signaling pathway. As several protein serine/threonine kinases play a positive regulatory role in TLR signaling, we focussed on protein serine/threonine phosphatases, the counterplayers of these kinases^19^. In this regard, the serine/threonine phosphatase PP2ACA has a well-established negative regulatory role in TLR mediated-NF-κB activation^28,29^. We reasoned that compromising the activity of PP2ACA should allow enhanced TLR-initiated responses. Accordingly, stable PP2ACA knock-down cells (H-17 PP2ACA^KD^) were generated (Fig. 4A and Supplementary Fig. S4A) and analyzed to monitor their reactivity in response to synthetic lipoproteins. In comparison to the cells transduced with the scrambled control shRNA, the pooled population of H-17 PP2ACA^KD^ cells showed a faster onset and higher magnitude of mCFP expression after stimulation with Pam3CSK4 (100 ng/mL) (Fig. 4B,C). Indeed, already after 2 h of stimulation with Pam3CSK4 (100 ng/mL), a significant increase in mCFP fluorescence could be detected by flow cytometry (Fig. 4B). Western Blotting demonstrated that a first increase in mCFP expression in H-17 PP2ACA^KD^ cells was already evident after 60 min of stimulation (Fig. 4D and Supplementary Fig. S4B), suggesting that the early detection of blue fluorescence by flow cytometry or microscopy is limited by fluorophore maturation of mCFP. Nevertheless, knock-down of PP2ACA clearly accelerated and elevated the response of this reporter cell population (Fig. 4D and Supplementary Fig. S4C-E), suggesting that this protein phosphatase is a suitable target to modulate TLR signaling in THP-1 cells.

### PP2ACA-knock-down H-17 cells are hypersensitive to bacterial lipoproteins and are useful for detecting mycoplasma contamination

**Figure 5:**
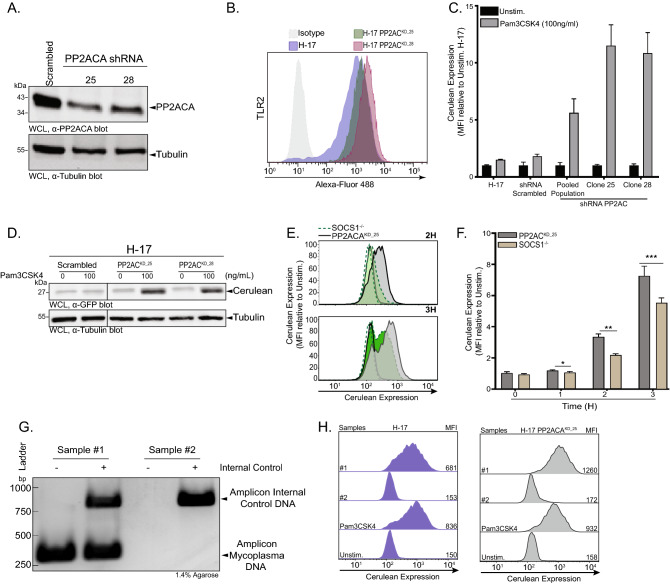
PP2ACA-knock-down H-17 cells are hypersensitive to bacterial lipoproteins and are useful for detecting mycoplasma contamination. (**A**) Western blots showing PP2ACA depletion in H-17 PP2ACA^KD^ clones 25 and 28 compare to H-17 cells. See also Supplementary Fig. S5A for uncropped, full-size original Western Blot. (**B**) Representative flow cytometry histograms for TLR2 from H-17, H-17 PP2ACA^KD_25^ and H-17 PP2ACA^KD_28^ and percentage of positive cells are indicated on corresponding panels. Histograms are normalised to the mode. (**C**) Quantification of mCFP expression in H-17 PP2ACA knock-down compared to scrambled upon stimulation with Pam3CSK4 (100 ng/ml) for 6 h. Bar graphs represent MFI ± SEM (n = 4). (**D**) Western blots showing increasing expression of mCFP in H-17 PP2ACA^KD_25^, H-17 PP2ACA^KD_25^ compared to H-17 Scrambled after treatment with Pam3CSK4 (100 ng/ml) for 2 h. See also Supplementary Fig. S5B for uncropped, full-size original Western Blot. (**E**) Representative flow cytometry histograms of mCFP expression in H-17 PP2ACA^KD_25^ [grey, solid line] compared to H-17 SOCS1^−/−^ [green, dashed line] after treatment for 2 h or 3 h with Pam3CSK4 (100 ng/ml). Histograms are normalised to the mode. Unfilled histograms represent unstimulated cells. (**F**) Quantification of mCFP expression in H-17 PP2ACA^KD^ compared to H-17 SOCS1^−/−^ after treatment for 1 h, 2 h or 3 h with Pam3CSK4 (100 ng/ml). Bar graphs represent MFI ± SEM (n = 4). (**G**) PCR analysis of a mycoplasma-positive (Sample #1) and a mycoplasma-negative (Sample #2) cell culture supernatant. See also Supplementary Fig. S5C for uncropped, full-size original agarose gel. (**H**) Representative flow cytometry histograms of mCFP expression in H-17 (left panels) or H-17 PP2ACA^KD_25^ cells (right panels) after 3 h of exposure to cell culture supernatants from (**G**). Incubation for 3 h with Pam3CSK4 (100 ng/ml) served as positive control. Histograms are normalised to the mode and the number indicates the mean fluorescent intensity (MFI).

To verify the increased signaling potential of PP2ACA^KD^ cells in response to lipoprotein, we derived single cell clones from the pooled population of H-17 PP2ACA^KD^ cells. Clones 25 and 28 (termed PP2ACA^KD_25^ and PP2ACA^KD_28^) showed about 90% reduction in PP2ACA expression as compared to H-17 treated with scrambled shRNA (Fig. 5A and Supplementary Fig. S5A). Flow cytometry demonstrated that PP2ACA^KD_25^ and PP2ACA^KD_28^ expressed slightly elevated TLR2 levels compared to the H-17 parent cell line, which could also increase their responsiveness towards lipoproteins (Fig. 5B). Indeed, PP2ACA^KD_25^ and H-17 PP2ACA^KD_28^ displayed elevated NF-κB-dependent production of the mCFP marker protein upon 3 h of stimulation with Pam3CSK4 (100 ng/mL) (Fig. 5C). Compared to the pooled population of shRNA-PP2AC-treated H17 cells, both clonal cell lines showed an almost doubling of the response (Fig. 5C). The elevated response by the PP2ACA knock-down cells was already obvious at an early time point after stimulation with lipoprotein (2 h), when flow cytometry as well as Western Blotting revealed a strong increase in mCFP expression (Fig. 5D and Supplementary Fig. S5B) again confirming that the lack of PP2ACA greatly accelerates TLR signaling. To examine, how the knock-down of PP2ACA compared to the deletion of SOCS1, H-17 PP2ACA^KD_25^ and H-17 SOCS1^−/−^ cells were stimulated with Pam3CSK4. As predicted, compromising PP2ACA, which acts within the TLR signaling cascade, lead to faster onset of mCFP expression compared to the deletion of SOCS1, which is involved in a negative feedback loop (Fig. 5E,F). These results demonstrate that stable depletion of PP2ACA accelerates reporter gene expression in response to a TLR ligand highlighting the potential to create ultrasensitive reporter cells.

Several *Mycoplasma* species are a frequent source of contamination in mammalian cell cultures, which often remain undected due to their slow growth, but which can corrupt experimental outcomes^30^. To underscore the usefulness of our reporter cell lines, we exposed H-17 cells and H-17 PP2ACA^KD_25^ cells to cell culture supernatants potentially containing *Mycoplasma*. Indeed, using PCR-based detection, we found mycoplasma contamination in the conditioned medium from one mammalian cell line recently imported into the laboratory (sample #1), while other cell lines were mycoplasma-negative (sample #2) (Fig. 5G and Supplementary Fig. S5C)). Importantly, when these conditioned media were added to the reporter cells, both H-17 cells as well as PP2ACA^KD_25^ cells showed a strong increase in mCFP fluorescence within 3 h in response to sample #1, while no mCFP expression was induced by the mycoplasm-free sample #2 (Fig. 5H). Moreover, mCFP expression by PP2ACA^KD_25^ cells in response to the mycoplasm-contaminated sample #1 was again doubled in comparison with H-17 cells (Fig. 5H). These results underscore the usefulness and high sensitivity of these reporter cells to detect frequent microbial contaminants in complex samples such as conditioned cell culture medium.

### PP1 inhibition potentiates the response of H-17 PP2ACA^KD^ towards lipoprotein

**Figure 6:**
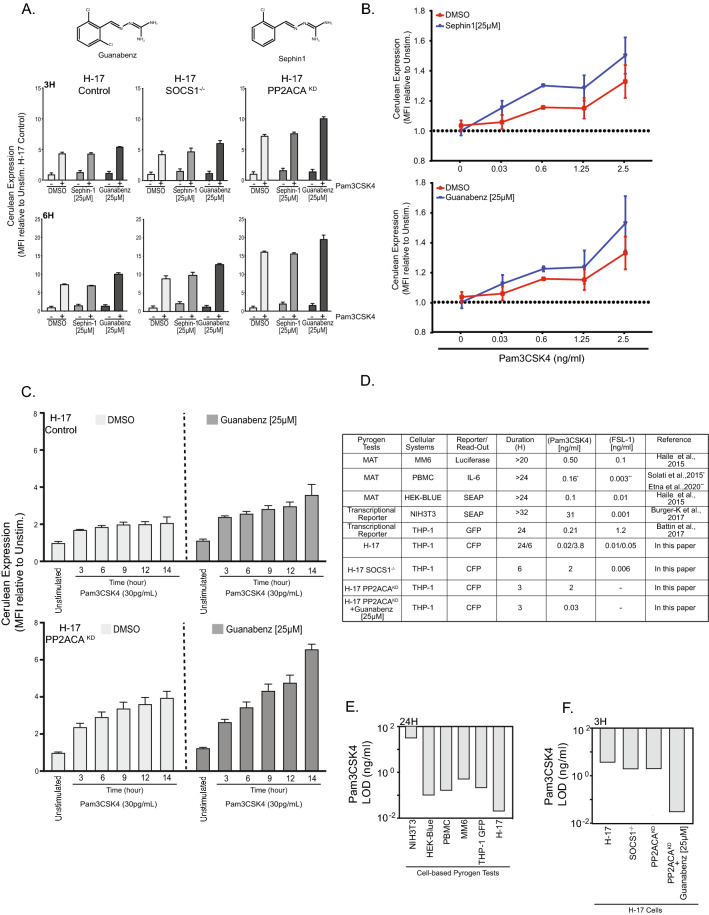
PP1 inhibition potentiates the response of H-17 PP2ACAKD towards lipoprotein. (**A**) H17 Control, H-17 SOCS1^−/−^, and H-17 PP2ACA^KD_25^ cells were stimulated with 100 ng/ml Pam3CSK4 in the presence of DMSO (solvent control), Sephin-1 (25 µM) or Guanabenz (25 µM). Cerulean expression was determined by flow cytometry 3 h (upper panel) or 6 h (Lower panel) after stimulation. Bars represent MFI ± SEM from three independent experiments. (**B**) Cells were treated as in (**A**) and stimulated for 6 h with the indicated concentrations of Pam3CSK4. Data points represent represent MFI ± SEM (n = 4). (**C**) H17 Mock or H-17 PP2ACA^KD^ were treated with DMSO or Guanabenz (25 µM) and stimulated with Pam3CSK4 (2 ng/ml) for 6, 9, 12 and 14 h respectively. Bar graphs represent MFI ± SEM (n = 4). (**D**) Comparison of different human cell-based pyrogen tests with regard to the limit of detection (LOD) for the TLR1/TLR2 ligand Pam3CSK4 or the TLR2/TLR6 ligand FSL-1. The respective read-out and the duration of stimulation is indicated. (**E**) LOD for Pam3CSK4 by different reporter cell lines upon 24 h of stimulation. LOD values were taken from references cited in (**D**). (**F**) Limit of detection (LOD) for Pam3CSK4 by H-17 reporter cells and derived knock-out and knock-down cell lines upon 3 h of stimulation.

Besides PP2A, also the protein phosphatase PP1, and in particular the PP1 regulatory subunit GADD34, has been proposed to limit TLR signaling^31,32^. Therefore, we wondered if small molecule inhibitors of GADD34, Guanabenz and Sephin-1^33,34^, could potentiate TLR signaling (Fig. 6A). To this end, the H-17-derived Control, the H-17 PP2ACA^KD^, and the H-17 SOCS1^−/−^ reporter cell lines were treated with Guanabenz (25 µM), Sephin-1 (25 µM), or solvent (DMSO) and then stimulated with 100 ng/ml Pam3CSK4 for 3 or 6 h. Interestingly, Guanabenz, but not Sephin-1, slightly increased the magnitude of the cellular response in every cell line tested (Fig. 6A). As observed before, H-17 PP2ACA^KD^ showed the most intense response with about 12-fold and 17-fold increase of mCFP fluorescence after 3 h or 6 h of stimulation, respectively (Fig. 6A). The treatment of H-17 PP2ACA^KD^ cells with Guanabenz or Sephin sensitized the cells to respond to Pam3CSK4 concentrations as low as 30 picogram/ml within 6 h (Fig. 6B). While Guanabenz treatment also resulted in a slightly elevated response of H-17 cells at these low concentrations of Pam3CSK4, PP1 inhibitor treatment further potentiated the hightened NF-κB reporter activity in the H-17 PP2ACA^KD^ cells over the course of 6–14 h (Fig. 6C). Direct comparison of the sensitivity and response kinetics of H-17 and H-17 PP2ACA^KD^ cells towards lipoproteins with other cellular pyrogen test systems revealed that Guanabenz-treated H-17 PP2ACA^KD^ cells are superior in two regards: on the one hand, these cells can detect lower conncentrations of TLR1/TLR2 and TLR2/TLR6 ligands compared to other suggested monocyte activation tests (Fig. 6D). Indeed, the limit of detection for Pam3CSK4 of the H-17 reporter cells, when measured after 24 h, is already lower than for any other cell-based test system (Fig. 6E). On the other hand, the H-17 cells, and more so the H-17 derived phosphatase knock-down or phosphatase-inhibited cells, show a dramatically accelerated response allowing clear recognition of pyrogen contamination within 3–6 h (Fig. 6C,F). Taken together, these results show that pharmacological inhibition of PP1 by Guanabenz can be used to further enhance celllular TLR-initiated responses. Accordingly, next-generation desginer cells have the potential for ultrasensitive detection of microbial impurities.

## Discussion

Cellular reporter systems, which respond to microbial contaminants, are not only useful for safety science, but also allow the detailed study and interrogation of innate immune pathways. Here, we present an optimized monocyte-based biosensor to detect and quantify TLR-triggered signaling. By genetic depletion and pharmacological inhibition of endogenous negative regulators of the TLR signalling cascade, the engineered cells show immediate, hypersensitive repsonses to microbe-derived components. Accordingly, our results validate several negative regulators of TLR signaling and provide a rationale framework for developing next-generation cell-based biosensors.

We first employed genetic disruption of the *SOCS1* gene, which encodes a key negative regulator of the TLR pathway^20^. SOCS1 is a transcriptional target of JAK-STAT-dependent cytokine-initiated signaling^35^. Upon TLR stimulation and NF-κB driven cytokine expression, secreted cytokines act in an autokrine manner to upregulate SOCS1^36^. SOCS1 is also an integral component of a negative feedback loop to limit TLR signaling as it can promote ubiquitination and proteasomal degradation of the TLR adapter protein Mal^24,37^. In line with this negative regulatory role, the deletion of SOCS1 resulted in a strong increase in NF-κB transcriptional activity in response to the TLR1/2 agonist Pam3CSK4. Most strikingly, expression of the reporter was also detected at a much earlier time point in the SOCS1^−/−^ cells. While in SOCS1-expressing cells the reporter gene became detectable 9–11 h after stimulation, clear upregulation of the reporter was already observed after 2–3 h stimulation in the SOCS^−/−^ cells. This unexpected finding indicates that in wildtype cells, SOCS1 is able to diminish TLR signaling right from the beginning. Accordingly, SOCS1 depletion does not only lead to elevated levels of NF-κB –dependent transcripts after prolonged TLR stimulation, as one would expect from its known involvement in a negative regulatory circuit, but SOCS1 also has a role in controling the initial activation of NF-κB. From a practical point of view, the ability to detect TLR-initiated signals much faster using SOCS1-deficient reporter cells already highlights the potential of our approach to target negative regulators of the pathway for achieving maximal reporter activity.

The gain-of-function observed upon depletion of negative regulatory elements is reminiscent of current checkpoint inhibitor approaches in tumor immunology. In that context, maximal T-cell responses against tumors are achieved by eliminating negative regulatory signaling in T cells^38,39^. Therefore, our approach differs from strategies, which aim to achieve a more sensitive detection of TLR agonists by overexpressing TLRs or their co-receptors. Such a strategy has been followed by Battin and colleagues, who expressed TLR4 together with MD2 and CD14 in THP-1 cells and could thereafter observe LPS-triggered transcriptional responses^15^. It is conceivable that a combination of TLR overexpression together with depletion of negative regulators in the same cell might offer maximal pathway output and could even further increase the sensitivity of such a reporter cell. However, such a dual manipulation with elevating the positive signal and reducing the negative counterregulation might also lead to enhanced background signaling in the absence of a stimulus, diminishing a potential net gain in signal to noise ratio. Efforts to overexpress particular TLRs or TLR co-receptors in THP-1 reporter cells might also be helpful to broaden the spectrum of recognized TLR agonists. Indeed, a major limitation of the THP-1-derived reporter cells developed in our study is their failure to respond to LPS. Previous reports have attributed the lack of LPS responsiveness by THP-1 cells to their low expression of CD14, a component of the CD14-TLR4-MD2 LPS signaling complex^40^. Accordingly, overexpression of CD14 in THP-1 cells could ameliorate this limitation turning the resulting reporter cells into a universal bio-detector of different microbial contaminants. Indeed, using NF-κB–driven gene expression as a read-out enables the sensitive detection of a wide range of stimuli and in principle, can report on canonical and non-canonical NF-κB signaling. On the other hand, the multitude of potential upstream agonists prohibits the direct identification of the microbial contaminant with this cellular reporter. Nevertheless, as the example of mycoplasma-contaminated cell cultures demonstrates, this does not limit the usefulness of this approach in practical terms, as the optimized reporter cells rapidly and unambiguously flag contaminated cell cultures.

An interesting aspect of our work is the confirmation that multiple protein phosphatases of the PPP family negatively regulate TLR signaling^19^. Indeed, PP2ACA can dephosphorylate several key components of the TLR-induced cascade including TAK-1, IKKα/β/γ or IRAK1, IκB-α, NF-κB or MAPKs^41–45^. Similar to our approach with SOCS1, we initially tried to generate a complete genetic knock-out of PP2AC via CRISPR/Cas9. However, in several independent attempts we were not able to obtain a single viable PP2AC-deficient clonal THP-1 cell line. As the catalytic subunit of this serine/threonine phosphatase can pair with different regulatory subunits to orchestrate a plethora of cellular functions, it appears that this enzyme is essential for cell viability and/or cell survival. Indeed, PP2AC knock-out mice die in utero early during embryonic development^46,47^. As an alternative, we generated PP2AC-deficient cells via RNAi-mediated knock-down. These cells maintained ~ 10% of PP2AC protein expression, remained viable, and showed the predicted gain-of-function with regard to TLR-stimulated NF-κB activity. Clearly, such a partial depletion or inhibition might be an appropriate approach to diminish other enzymes with negative regulatory roles in the TLR signaling cascade. Indeed, pharmacological inhibitors to temporarily block PP1 activity, another protein phosphatase involved in TLR signaling was able to elevate TLR responses and this PP1 inhibition synergized with PP2AC depletion, generating the most sensitive cell-based detector for lipoproteins reported to date.

Besides the increased sensitivity, a major benefit of our genetic manipulation of the THP-1 cells from a practical point of view is the accelerated appearance of the transcriptional reporter (in our case the blue-fluorescent mCFP protein). The rapid increase in mCFP levels could dramatically speed up the detection of pyrogenic impurities by cellular systems and would make these systems more competitive to the widely used and regulatory approved limulus amoebocyte lysate (LAL) test. Moreover, as genetic and pharmocological treatments can easily be combined to target additional protein phosphatases, but potentially also other enzymes involved in counterregulation of the TLR signaling cascade, such as de-ubiquiniating enzymes, a further optimization of reporter cells appears feasible. It can be envisioned that such optimized next-generation reporter cells are poised to serve as the cellular monitoring systems of choice to detect pyrogenic contaminants along the drug manufacturing process, thereby constituting an integral element in producing safe therapeutics.

## STAR methods

### Reagents, cell lines and cell culture

The human monocytic cell line THP-1 was maintained in RPMI 1640 supplemented with 10% fetal calf serum (FCS). The Human embryonic kidney 293 cells were maintained in DMEM supplemented with 10% calf serum. Cells were cultured in a humidified atmosphere with 5% CO_2_ at 37˚C. All materials including standard LPS (Escherichia coli 0127:B8) and Phorbol-12-myristate-13-acetate (PMA) were obtained from Sigma-Aldrich (St. Louis, MO) unless stated otherwise. Agonists for TLR1/2 (Pam3CSK4, synthetic triacylated lipopeptide), TLR2/6 (FSL-1, synthetic diacylated lipopeptide), TLR3 (poly (I:C), synthetic analogue of double-stranded RNA (dsRNA)), TLR4 (LPS-EB ultrapure), TLR5 (Flagellin), TLR7 (Gardiquimod, imidazoquinoline analogue) and TLR9 (Class B CpG oligonucleotide ODN 2006) were purchased from Invivogen (San Diego, CA). Recombinant TNF-α protein was purchased from Peprotech (London, UK). Genomic DNA from *Neisseria gonorrhoeae* was provided by P. Muenzner^48^.

### Whole bacteria lysate preparation

About 10^7^ *E. coli K12 (Nova Blue)* were suspended in 200 µl PBS and subjected to four cycles of freeze (in liquid nitrogen) and thaw (37 °C). Afterwards, the mixture was sonicated three times for 30 s, boiled for 5 min, aliquoted and stored at − 80 °C for subsequent use.

### Recombinant DNA

Reporter constructs were generated from pHAGE-NF-κB-TA-fLuciferase-UBC-tdTomatoW (pHAGE) provided by Darell Kotton (addgene plasmid #49335)^26^. Using primers 5′-ATAACTAGTATAGGTACCATAGCTAGCATAACCGGTATAGCCACCATGGAAGACGCCAAA-3′ and 5′-CGGATCCTTACACGGCGATCTT-3′ we amplified the MCS-TA-fLuciferase-UBC-tdTomato cassette and inserted this amplicon back into the SpeI / BamHI restriction sites of pHAGE, essentially elimating the NF-κB binding sites generating 0xκB-TA_P_-FLuci.

To generate the pHAGE- NF-κB-TA-mCerulean-UBC-tdTomatoW (4xκB-TA_P_-mCFP), the mCerulean cDNA was amplified from the plasmid pmCerulean-C1 (kind gift of D. Piston, Vanderbilt University Medical Center, Nashville,TN) using primers mCFP_sense: 5′-CGGTACTGTTGGTAAAGCCACCATGGTGAGCAAGGGCGAGGAGCTGTTCA-3′ and mCFP_anti: 5′-TATGGATCCTTATTTGTACAGTTCGTCCAT-3′ while the 4 × kappa binding site DNA sequence was amplified from pHAGE using primers kappa_site_sense 5′-ATAGACTAGTATAACCGGTATAGCTAGCCTAGTGGGAATTTCCGGGAAT-3′ and kappa_site_anti 5′-TGAACAGCTCCTCGCCCTTGCTCACCATGGTGGCTTTACCAACAGTACCG-3′. The two PCR amplicons were fused by Splicing by overlapping Extension (SOEing) PCR using primers kappa_site_sense and mCFP_anti and the resulting fusion product was inserted into the SpeI/BamHI restriction sites of pHAGE essentially replacing the NF-κB-TA-fLuciferase part. In a similar manner, the 0xκB-TA_P_-mCFP construct was generated by inserting the mCerulean cDNA (amplified with primers mCFP_SpeI_sense 5′-ATAACTAGTGGTACCGCTAGCACCGGTGCCACCATGGTGAGCAAGGGCGAGGAGC-3′ and mCFP_anti) into the SpeI/BamHI sites of pHAGE.

To generate the pHAGE-8κB-TA-mCerulean-UBC-tdTomatoW (8xκB-TA_P_-mCFP), synthetic oligonucleotides encompassing four canonical kappa binding sites (4xkappa_sense 5′-CTAGTGGGAATTTCCTCTGATGGGAATTTCCCTCGACGGGAATTTCCCTCGACGGGAATTTCCA-3′ and 4xkappa_anti 5′-CCGGTGGAAATTCCCGTCGAGGGAAATTCCCGTCGAGGGAAATTCCCATCAGAGGAAATTCCCA-3′ were annealed and inserted into the SpeI and AgeI restriction sites of 4xκB-TA_P_-mCFP resulting in 8xκB-TA_P_-mCFP.

### sgRNA constructs and cloning

Single guide RNA (sgRNAs) targeting tdTomato and SOCS1 were designed for optimal depletion using the CHOP-CHOP algorithm (http://chopchop.cbu.uib.no/)^49^ and CRISPinatoR (https://crispinator.com)^50^. The selected oligos with BsmGI overhangs were synthesized and annealed. For targeting SOCS1, sgSOCS1_sense 5′-AAACTTCCCGAATGACTCGACACGC-3′ and sgSOCS1_anti 5′-CACCGCGCTGCCGGTCAAATCTGGAAGG-3′ were used. For tdTomato, the oligo sgTomato_sense 5′- CACCGTGGAGCCGTACATGAACTGG-3′ and sgTomato_anti 5′-AAACCCAGTTCATGTACGGCTCCAC-3′ were used. The annealed double-stranded oligos were inserted into the BsmGI site of lentiCRISPR v2 (gift from Feng Zhang; Addgene plasmid #52961). Plasmids were confirmed by sequencing.

### RNA interference by small hairpin RNA

For shRNA-expressing lentiviral particles, the vector pLKO.1 developed by Stewart and colleagues^51^ was applied. The different shRNAs were designed by using the AAN19 algorithm and shRNA selection program of the Whitehead Institute for Biomedical Research (http://sirna.wi.mit.edu/). As a control, Scrambled_shRNA sense 5′-CCGGGGTGTCTCAGGCACTTATATTCTCGAGAATATAAGTGCCTGAGACACCTTTTTG-3′ and Scrambled_shRNA_anti 5′-AATTCAAAAAGGTGTCTCAGGCACTTATATTCTCGAGAATATAAGTGCCTGAGACACC-3′ were used, for the PP2ACA knock-down PP2A_shRNA_sense 5′-CCGGAATGGGAAGAGCAACAGTAACCTCGAGGTTACTGTTGCTCTTCCCATTTTTTTG-3′ and PP2A_shRNA_anti 5′-AATTCAAAAAAATGGGAAGAGCAACAGTAACCTCGAGGTTACTGTTGCTCTTCCCATT-3′ were used. The primers were annealed and cloned into AgeI and EcoRI restriction sites of pLKO.1. The insertion of the shRNA cassette was verified by sequencing.

### Lentiviral particle production

Lentiviral particles were produced as previously described^52^. Briefly, HEK293T cells were transfected by calcium precipitation with three plasmids (pLKO.1 containing shRNA or sgRNA, psPAX and pMD2.G). Seventy-two hours post-transfection, lentiviral particles were harvested and were either used freshly or concentrated by ultracentrifugation (20,000 rpm, 130 min at 4 °C) in 20% sucrose gradient. Concentrated viruses were redissolved in 1% BSA and stored at − 80 °C for subsequent use.

### Generation of stable cell lines

Approximately 1 * 10^6^ THP-1 cells were transduced by spinfection. Fresh supernatant or concentrated virus was added to cells in the presence of polybrene (8 µg/ml) and centrifuged at 800 rpm for 1 h at 28 °C. For tdTomato-based selection of pHAGE-transduced cells, single tdTomato-positive cells were sorted into 96-well plates 5 days post-transduction (FACSAriaIII). For lentiCRISPR knock-out cells, H-17 cells were transduced with a combination of sgRNAs against SOCS1 and tdTomato, single tdTomato-negative cells were selected 14 days after transduction and sorted into 96-well plates. In the case of shRNA-mediated knock-down, transduced cells were selected with 1 µg/ml puromycin for 7 days and single cells were diluted manually in 96-well plates. Clonal cell lines were expanded from single cells in 50% conditioned medium and at least two independent clonal cell lines were further analysed.

### Cerulean reporter assay

For Cerulean reporter assays, THP-1 cerulean reporter cells were starved overnight and incubated in the presence of stimuli for 6 h. Assays were performed in 96-well round bottom plates at 1 × 10^5^ cells per well in a total volume of 200 μl (including stimulus). Cells were then analyzed by flow cytometry. Mean and standard deviation of the geometric mean of fluorescence intensity (gMFI) of the viable population of reporter cells was determined. All samples were analysed in triplicates, unless indicated otherwise. Furthermore, fluorescent protein expression was evaluated by fluorescence microscopy.

### Detection of mycoplasm contamination

Cell culture supernatants from mycoplasm-contaminated or mycoplasm-free cell lines were collected after 14 days of continuous culture and stored at − 20 °C in sterile DNA-free microcentrifuge tubes. Upon thawing, samples were heated (10 min at 95 °C), centrifuged (5 min at 13,000 rpm), and the cleared supernatant was collected. For mycoplasm detection by H-17 reporter cells, 20 µl of the cleared supernatant was added to H-17 cells in 180 µl of medium in 96-well plates. In addition, mycoplasm contamination was analysed by PCR as described^53^. In brief, 1 µl of the cleared culture supernatant was analysed with a mix of PCR primers (sense-1 5′-CGCCTGAGTAGTACGTWCGC-3′, sense-2 5′-TGCCTGRGTAGTACATTCGC-3′, sense-3 5′-CGCCTGAGTAGTATGCTCGC-3′, sense-4 5′-CGCCTGGGTAGTACATTCGC-3′, antisense 5′-GCGGTGTGTACAARMCCCGA -3′) targeting the 16S rRNA coding genes of several Mycoplasm species. PCR conditions were as follows: 2 min 94 °C for 1 cycle, 15 s 94 °C, 20 s 60 °C, 1 min 72 °C for 30 cycles, 10 min 72 °C. As an internal control, the cloned 16S-rRNA gene of *Acholeplasma laidlawii* containing an internal 476 bp stuffer was used^54^. This internal control results in a 986 bp fragment, when using the above PCR conditions.

### Flow cytometry

Flow cytometry samples were prepared in FACS buffer (PBS pH 7.4 + 1% heat inactivated FCS). Acquisition was performed using FACSFortessa or FACSVerse (both BD Biosciences, San Jose, CA). Fluorescence intensity is shown on a standard logarithmic scale. The y-axis displays cell counts (minimum 5000/sample) normalized to mode.

### Whole cell lysate and immunoblotting

For whole cell lysates, cells were lysed in NF-κB cell lysis buffer (10 mM Tris, pH 7.4, 100 mM NaCl, 1 mM EDTA, 1 mM EGTA, 1 mM NaF, 20 mM Na_4_P_2_O_7_, 2 mM Na_3_VO_4_, 0.1% SDS, 0.5% sodium deoxycholate, 1% Triton-X 100, 10% glycerol) supplemented with protein inhibitors Leupeptin, Aprotinin, Benzamidin, and Pefabloc. For immunoblotting, whole cell lysates were separated by 10% SDS-PAGE, transferred to PVDF membranes, blocked with BSA, probed with primary antibodies at 4 °C over night and developed using enhanced chemiluminescence (ChemiDoc; BioRad).

### Statistical analysis

Flow cytometry data were processed in FlowJo v10 and are presented as geometric mean ± SEM from triplicate samples. Comparisons were statistically tested using Two-way ANOVA test. *P* values < 0.05 were considered to be statistically significant. All graphs and statistical tests were performed in GraphPad Prism 9.0.

## Supplementary Information

Supplementary Information.

## References

[1] Janeway CA, Medzhitov R (2002). Innate immune recognition. Annu. Rev. Immunol..

[2] Girardin SE (2003). Nod1 detects a unique muropeptide from gram-negative bacterial peptidoglycan. Science.

[3] Rehwinkel J, Gack MU (2020). RIG-I-like receptors: Their regulation and roles in RNA sensing. Nat. Rev. Immunol..

[4] Hopfner KP, Hornung V (2020). Molecular mechanisms and cellular functions of cGAS-STING signalling. Nat. Rev. Mol. Cell. Biol..

[5] Akira S, Takeda K, Kaisho T (2001). Toll-like receptors: Critical proteins linking innate and acquired immunity. Nat. Immunol..

[6] Medzhitov R, Horng T (2009). Transcriptional control of the inflammatory response. Nat. Rev. Immunol..

[7] Lu YC, Yeh WC, Ohashi PS (2008). LPS/TLR4 signal transduction pathway. Cytokine.

[8] Evans SS, Repasky EA, Fisher DT (2015). Fever and the thermal regulation of immunity: The immune system feels the heat. Nat. Rev. Immunol..

[9] Hartung T (2021). Pyrogen testing revisited on occasion of the 25th anniversary of the whole blood monocyte activation test. Altex.

[10] Hartung T, Wendel A (1995). Detection of Pyrogens using human whole blood. Altex.

[11] Fennrich S (1999). Detection of endotoxins and other pyrogens using human whole blood. Dev. Biol. Stand..

[12] Hoffmann S (2005). International validation of novel pyrogen tests based on human monocytoid cells. J. Immunol. Meth..

[13] O'Neill LA, Golenbock D, Bowie AG (2013). The history of Toll-like receptors—redefining innate immunity. Nat. Rev. Immunol..

[14] Zhang Q, Lenardo MJ, Baltimore D (2017). 30 years of NF-kappaB: A blossoming of relevance to human pathobiology. Cell.

[15] Battin C (2017). A human monocytic NF-kappaB fluorescent reporter cell line for detection of microbial contaminants in biological samples. PLoS ONE.

[16] Kondo T, Kawai T, Akira S (2012). Dissecting negative regulation of toll-like receptor signaling. Trends Immunol..

[17] Fitzgerald KA, Kagan JC (2020). Toll-like receptors and the control of immunity. Cell.

[18] Hamerman JA (2016). Negative regulation of TLR signaling in myeloid cells–implications for autoimmune diseases. Immunol. Rev..

[19] Seumen CHT, Grimm TM, Hauck CR (2021). Protein phosphatases in TLR signaling. Cell Commun. Signal.

[20] Kinjyo I (2002). SOCS1/JAB is a negative regulator of LPS-induced macrophage activation. Immunity.

[21] Horwood NJ (2006). Bruton's tyrosine kinase is required for TLR2 and TLR4-induced TNF, but not IL-6, production. J. Immunol..

[22] Sakuma C, Sato M, Takenouchi T, Kitani H (2014). Specific binding of the WASP N-terminal domain to Btk is critical for TLR2 signaling in macrophages. Mol. Immunol..

[23] Schmaler M (2009). Lipoproteins in Staphylococcus aureus mediate inflammation by TLR2 and iron-dependent growth in vivo. J. Immunol..

[24] Mansell A (2006). Suppressor of cytokine signaling 1 negatively regulates Toll-like receptor signaling by mediating Mal degradation. Nat. Immunol..

[25] Honda K, Taniguchi T (2006). IRFs: Master regulators of signalling by Toll-like receptors and cytosolic pattern-recognition receptors. Nat. Rev. Immunol..

[26] Wilson AA (2013). Lentiviral delivery of RNAi for in vivo lineage-specific modulation of gene expression in mouse lung macrophages. Mol. Ther..

[27] Strebovsky J, Walker P, Lang R, Dalpke AH (2011). Suppressor of cytokine signaling 1 (SOCS1) limits NFkappaB signaling by decreasing p65 stability within the cell nucleus. FASEB J..

[28] Shanley TP, Vasi N, Denenberg A, Wong HR (2001). The serine/threonine phosphatase, PP2A: Endogenous regulator of inflammatory cell signaling. J. Immunol..

[29] Sun L (2017). Myeloid-specific gene deletion of protein phosphatase 2A magnifies MyD88- and TRIF-dependent inflammation following endotoxin challenge. J. Immunol..

[30] Drexler HG, Uphoff CC (2002). Mycoplasma contamination of cell cultures: Incidence, sources, effects, detection, elimination, prevention. Cytotechnology.

[31] Li HY (2008). Deactivation of the kinase IKK by CUEDC2 through recruitment of the phosphatase PP1. Nat. Immunol..

[32] Gu M (2014). Phosphatase holoenzyme PP1/GADD34 negatively regulates TLR response by inhibiting TAK1 serine 412 phosphorylation. J. Immunol..

[33] Lin W (2008). Enhanced integrated stress response promotes myelinating oligodendrocyte survival in response to interferon-gamma. Am. J. Pathol..

[34] Das I (2015). Preventing proteostasis diseases by selective inhibition of a phosphatase regulatory subunit. Science.

[35] Starr R (1997). A family of cytokine-inducible inhibitors of signalling. Nature.

[36] Fujimoto M, Naka T (2010). SOCS1, a negative regulator of cytokine signals and TLR responses, in human liver diseases. Gastroenterol. Res. Pract..

[37] Baetz A, Frey M, Heeg K, Dalpke AH (2004). Suppressor of cytokine signaling (SOCS) proteins indirectly regulate toll-like receptor signaling in innate immune cells. J. Biol. Chem..

[38] Pardoll DM (2012). The blockade of immune checkpoints in cancer immunotherapy. Nat. Rev. Cancer.

[39] Waldman AD, Fritz JM, Lenardo MJ (2020). A guide to cancer immunotherapy: From T cell basic science to clinical practice. Nat. Rev. Immunol..

[40] Bosshart H, Heinzelmann M (2016). THP-1 cells as a model for human monocytes. Ann. Transl. Med..

[41] Dobierzewska A, Giltiay NV, Sabapathi S, Karakashian AA, Nikolova-Karakashian MN (2011). Protein phosphatase 2A and neutral sphingomyelinase 2 regulate IRAK-1 protein ubiquitination and degradation in response to interleukin-1beta. J. Biol. Chem..

[42] Sun L (2007). Tristetraprolin (TTP)-14-3-3 complex formation protects TTP from dephosphorylation by protein phosphatase 2a and stabilizes tumor necrosis factor-alpha mRNA. J. Biol. Chem..

[43] Li S, Wang L, Berman MA, Zhang Y, Dorf ME (2006). RNAi screen in mouse astrocytes identifies phosphatases that regulate NF-kappaB signaling. Mol. Cell..

[44] Kray AE (2005). Positive regulation of IkappaB kinase signaling by protein serine/threonine phosphatase 2A. J. Biol. Chem..

[45] Yang J, Fan GH, Wadzinski BE, Sakurai H, Richmond A (2001). Protein phosphatase 2A interacts with and directly dephosphorylates RelA. J. Biol. Chem..

[46] Gotz J, Probst A, Ehler E, Hemmings B, Kues W (1998). Delayed embryonic lethality in mice lacking protein phosphatase 2A catalytic subunit Calpha. Proc. Natl. Acad. Sci. U. S. A..

[47] Gu P, Qi X, Zhou Y, Wang Y, Gao X (2012). Generation of Ppp2Ca and Ppp2Cb conditional null alleles in mouse. Genesis.

[48] Muenzner P, Hauck CR (2020). *Neisseria gonorrhoeae* blocks epithelial exfoliation by nitric-oxide-mediated metabolic cross talk to promote colonization in mice. Cell Host Microbe.

[49] Montague TG, Cruz JM, Gagnon JA, Church GM, Valen E (2014). CHOPCHOP: A CRISPR/Cas9 and TALEN web tool for genome editing. Nucleic Acids Res..

[50] Tuladhar R (2019). CRISPR-Cas9-based mutagenesis frequently provokes on-target mRNA misregulation. Nat. Commun..

[51] Stewart SA (2003). Lentivirus-delivered stable gene silencing by RNAi in primary cells. RNA.

[52] Grimm TM, Dierdorf NI, Betz K, Paone C, Hauck CR (2020). PPM1F controls integrin activity via a conserved phospho-switch. J. Cell. Biol..

[53] Uphoff CC, Drexler HG (2005). Detection of mycoplasma contaminations. Methods Mol. Biol..

[54] Uphoff CC, Drexler HG (2002). Comparative PCR analysis for detection of mycoplasma infections in continuous cell lines. In Vitro Cell. Dev. Biol..

