# Supplementary material for: Elimination of negative feedback in TLR signalling allows rapid and hypersensitive detection of microbial contaminants
Source: Sci Rep. 2021 Dec 24;11:24414. doi: 10.1038/s41598-021-03618-9 (PMC8709846; doi:10.1038/s41598-021-03618-9)
Supplement: Supplementary file 1 — Supplementary Information. [file 41598_2021_3618_MOESM1_ESM.pdf]

Supporting information

Elimination of negative feedback in TLR signalling allows rapid and hypersensitive detection of microbial contaminants

Authors

Clovis H. T. Seumen[1,2], Urte Tomasiunaite[1], Daniel F. Legler[2,3] Christof R. Hauck[1,2,4*]

Affiliations

[1]Lehrstuhl für Zellbiologie, Fachbereich Biologie, Universität Konstanz, 78457 Konstanz, Germany

[2]Kooperatives Promotionskolleg "InViTe“, Universität Konstanz, 78457 Konstanz

[3]Biotechnology Institute Thurgau (BITg) at the University of Konstanz, Kreuzlingen, Switzerland

[4]Konstanz Research School Chemical Biology, Universität Konstanz, 78457 Konstanz, Germany

Figure S1:

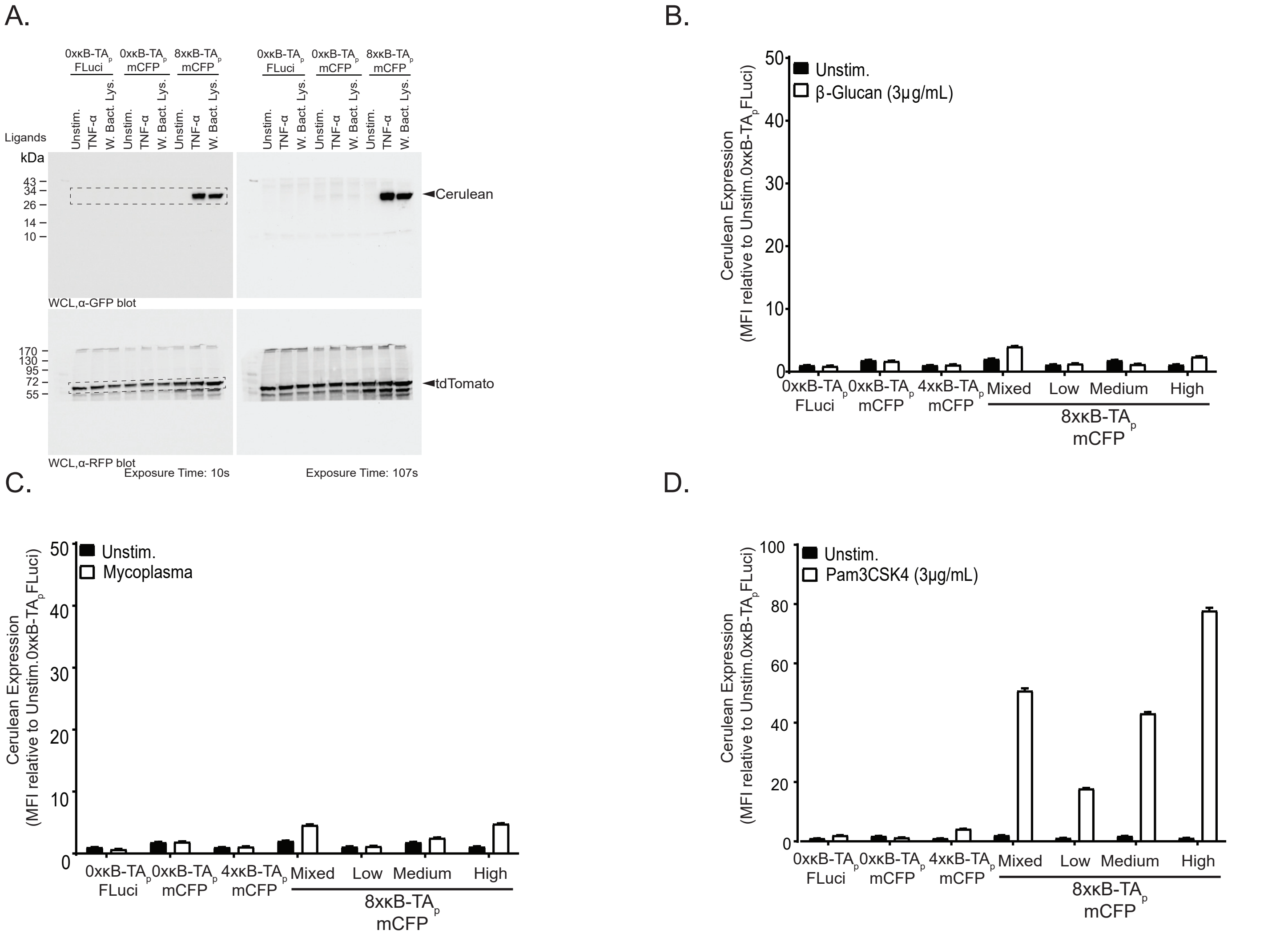

Figure S1: **Characterisation of 8xκB-TA$_p$mCFP cells. Related to figure 1**
A.Uncropped western blot images from figure 1D. Dashed boxes indicate cropped area used in the main figure.
B. Quantification of mCFP in mixed THP-1 cells, Low, medium and high tdTomato expressing cells respectively after stimulation with β-glucan (3μg/ml) for 24 H. The other three constructs are used as control. Bar graphs show MFI±SEM (n = 2)
C. Quantification of mCFP in mixed THP-1 cells, Low, medium and high tdTomato expressing cells respectively after stimulation with mycoplasma (3μg/ml) for 24 H. The other three constructs are used as control. Bar graphs show MFI±SEM (n = 2)
D. Quantification of mCFP in mixed THP-1 cells, Low, medium and high tdTomato expressing cells respectively after stimulation with Pam3CSK4 (3μg/ml) for 24 H. The other three constructs are used as control. Bar graphs show MFI±SEM (n = 2)

Figure S2:

A.

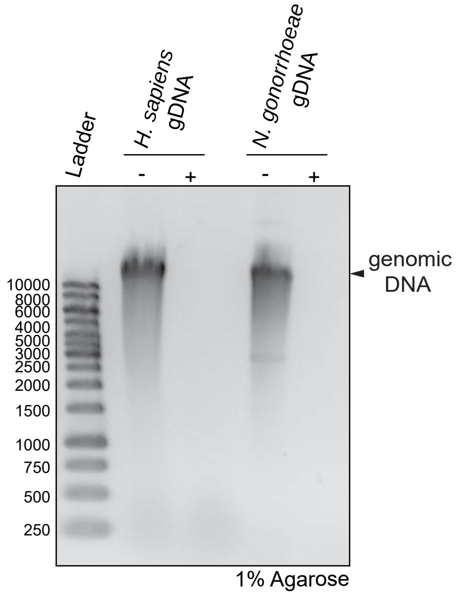

B.

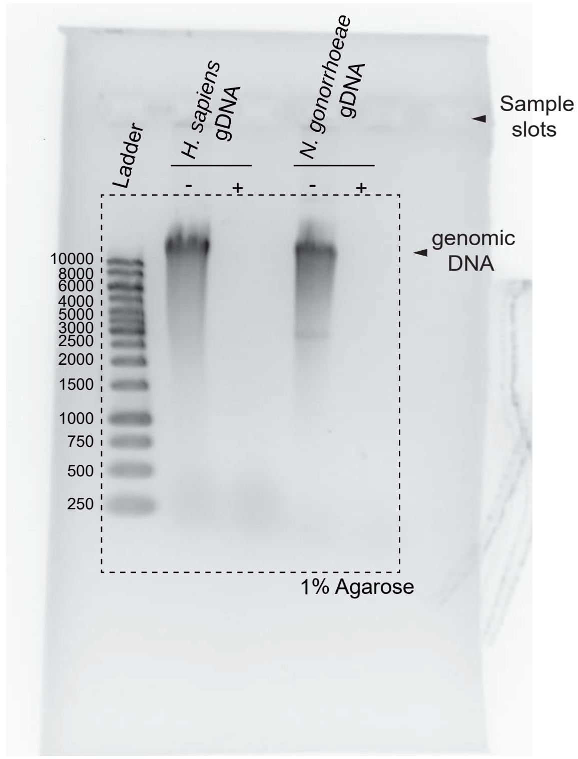

C.

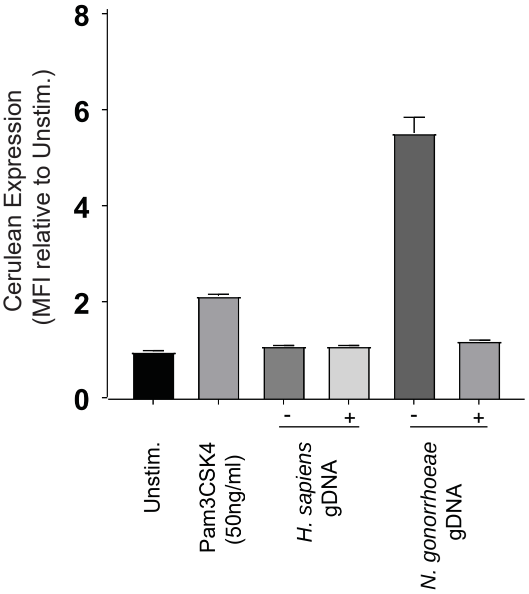

Figure S2: H-17 Cells detects gonoccocal genomic DNA. Related to figure 2

A. Agarose gel electrophoresis showing digestion of *N. gonorrhoeae* genomic DNA with DNase I compare to Human genomic DNA.

B. Uncropped Agarose gel images from figure S2A. Dashed boxes indicate dropped area used in the main figure.

C.Quantification of mCFP in H-17 cells stimulated with either *N. gonorrhoeae* genomic DNA or *H. sapiens* for 6 hours, digested and undigested respectively. Bar graphs show median fluorescent intensity (MFI) ± SEM. (n = 3)

Figure S3

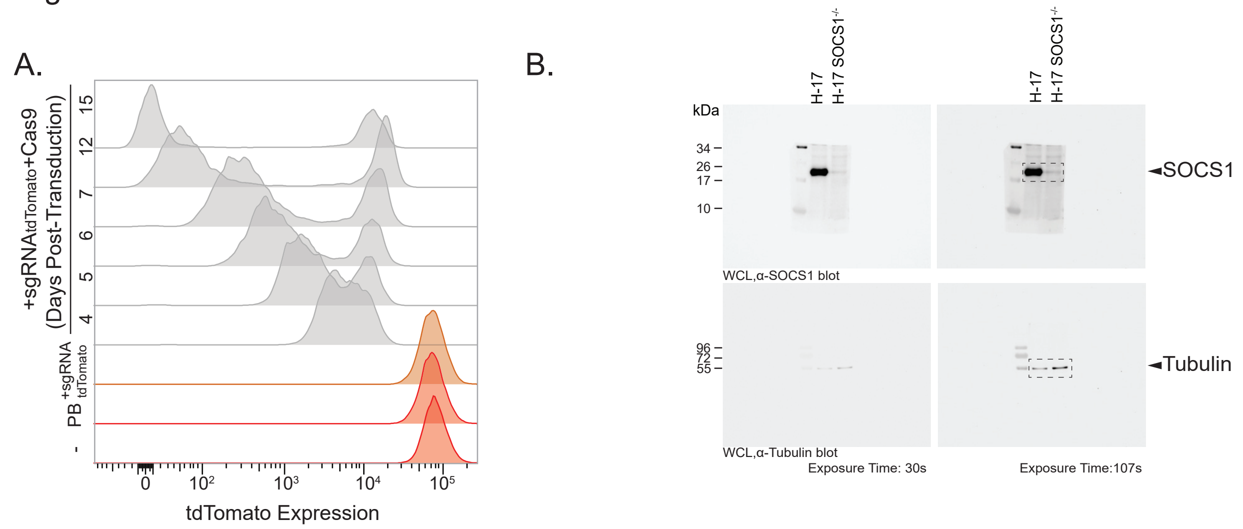

Figure S3: tdTomato depletion kinetic. Related to figure 3

A. Representative flow cytometry histograms for tdTomato depletion kinetic in H-17 cells after transduction with lentivirus carrying sgRNA against tdTomato alone and the lentivirus carrying Cas9 alone. dTomato levels are monitored for 15 days' post-transduction.

Cells treated with polybrene (PB) and lentivirus carrying sgRNA against tdTomato alone served as controls. Histograms are normalised to the mode.

B.Uncropped western blot gel images from figure 3B. Dashed boxes indicate cropped area used in the figure.

Figure S4:

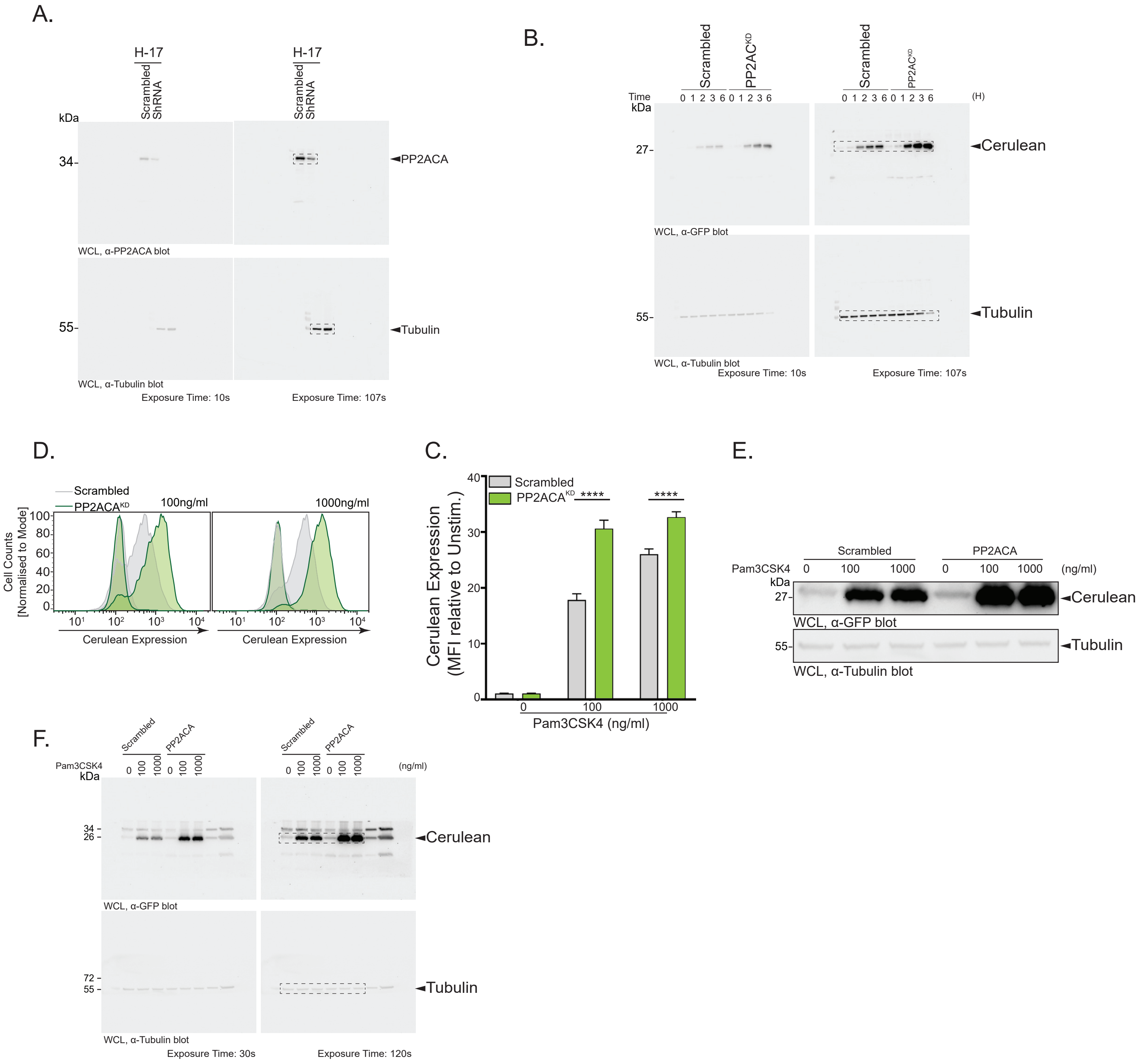

Figure S4: H-17 PP2ACA knock-down cells displays better response compared to H-17 Scrambled. Related to figure 4

A.Uncropped western blot gel images from figure 4A. Dashed boxes indicate dropped area used in the main figure.

B.Uncropped western blot gel images from figure 4D. Dashed boxes indicate dropped area used in the main figure.

C. Representative flow cytometry histograms of mCFP expression in H-17 PP2ACA^{KD} compared to scrambled H-17 after treatment for 6 hours with Pam3CSK4 100ng/ml and 1000ng/mL respectively.

Histograms are normalised to the mode.

D. Quantification of mCFP expression in H-17 PP2ACA^{KD} compared to scrambled H-17 after treatment for 6 hours with Pam3CSK4 100ng/ml and 1000ng/ml respec-tively. Two-way ANOVA shows significant difference between both cell types (**p < 0.01, ***p < 0.001, ****p < 0.001).Bar graphs represent MFI±SEM. (n = 4)

E. Immunoblot of mCFP expression in H-17 PP2ACA^{KD} compared to scrambled H-17 after treatment for 6 hours with Pam3CSK4 100ng/ml and 1000ng/ml respectively.

F. Uncropped western blot gel images from figure S4E. Dashed boxes indicate dropped area used in the main figure.

Figure S5:

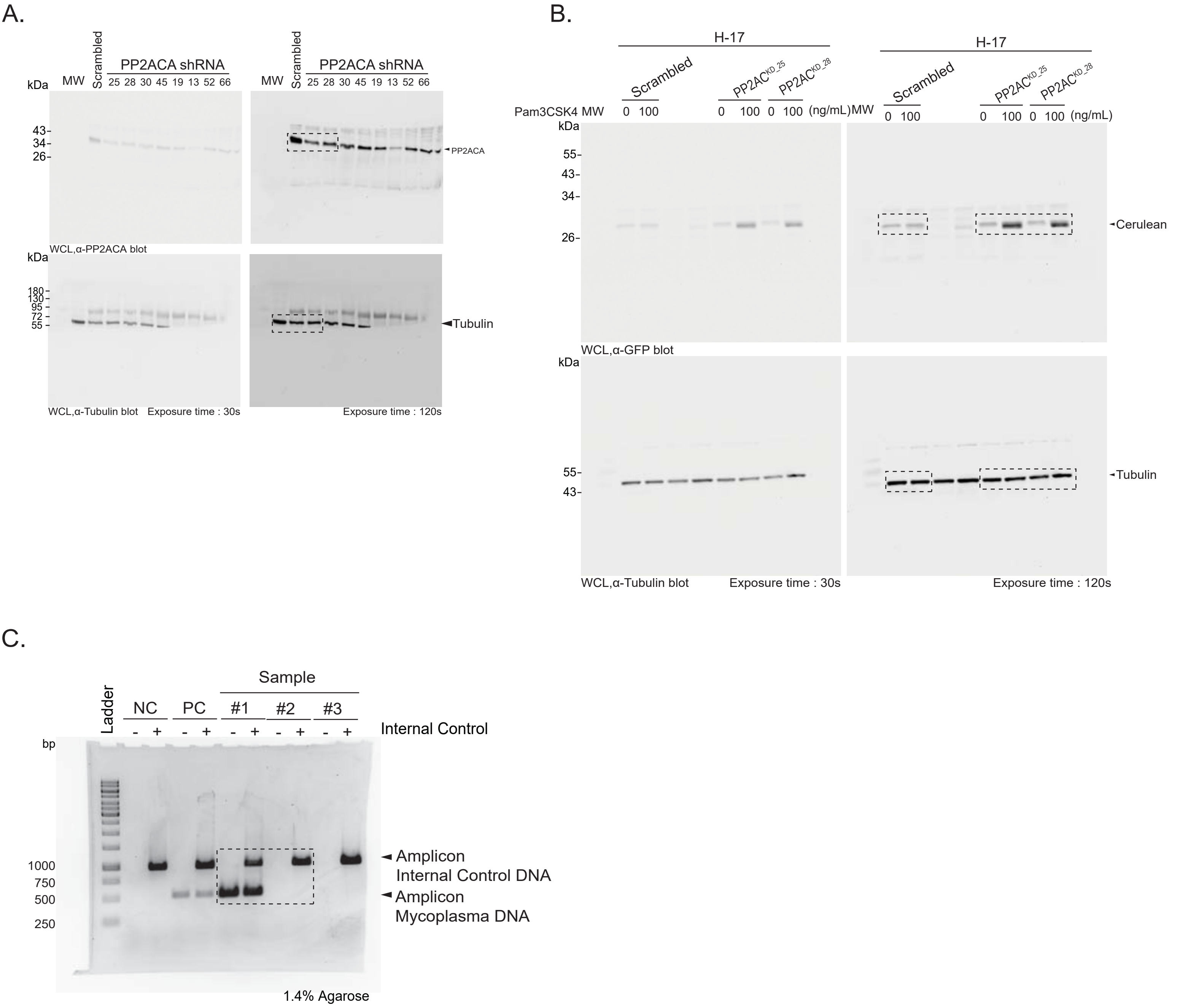

Figure S5: Related to figure 5

A. Uncropped western blot gel images from figure 5A. Dashed boxes indicate dropped area used in the main figure.

B. Uncropped western blot gel images from figure 5D. Dashed boxes indicate dropped area used in the main figure.

C. Uncropped agarose gel images from figure 5G. Dashed boxes indicate dropped area used in the main figure.
